# Gallery Architecture and Reproductive Strategy of *Ips hauseri* (Coleoptera: Curculionidae) in a *Picea schrenkiana* Forest: Implications for Population Dynamics Under Outbreak Conditions

**DOI:** 10.3390/insects17030238

**Published:** 2026-02-25

**Authors:** Yihao Fan, Lulu Dai, Haiming Gao

**Affiliations:** 1 College of Forestry and Landscape Architecture, Xinjiang Agricultural University, Urumqi 830052, China; fyhxjau@163.com; 2 Co-Innovation Center for Sustainable Forestry in Southern China, College of Forestry, Nanjing Forestry University, Nanjing 210036, China; dailulu@njfu.edu.cn

**Keywords:** bark beetles, gallery development, mating reproduction, population density, intraspecific competition

## Abstract

Outbreaks of *Ips hauseri*, a major bark beetle pest in Central Asian *Picea schrenkiana* forests, have intensified under climate warming and prolonged droughts. However, the reproductive behavior and gallery construction strategies of this species remain poorly understood, limiting our ability to predict its population dynamics. Here, we dissected 219 galleries from infested spruce trees in Hami, Xinjiang, during an outbreak period (2024–2025). We identified 11 distinct gallery morphologies, with harem size (number of females per male) ranging from one to seven. Gallery length was positively correlated with egg production. Reproductive output peaked at a harem size of five, beyond which both gallery dimensions and fecundity declined. Host tree diameter at breast height (DBH) significantly influenced gallery complexity, with larger trees supporting more maternal galleries. Upward-oriented galleries were longer and contained more eggs than downward ones. Intraspecific competition, mediated by gallery adjacency and spatial orientation, strongly affected offspring development. Our results demonstrate that *I. hauseri* exhibits flexible gallery architecture and reproductive adjustments in response to resource availability and competition—a behavioral plasticity that likely contributes to its outbreak potential. Monitoring gallery morphology and harem size could enhance early detection and population forecasting for this increasingly damaging forest pest.

## Simple Summary

*Ips hauseri* has long posed a threat to the health of coniferous forests in Central Asia. Since 2020, this pest has caused severe damage in the Hami forest region. However, its cryptic behavior makes it difficult to directly observe the complete reproductive and developmental process associated with gallery construction. Therefore, we systematically dissected its gallery-building process in stages. This study investigates the diverse types of gallery structures and their corresponding adult population dynamics at different developmental time points, examines reproductive development within various gallery structures, and compares the influence of different factors on gallery development. The aim is to gain a more detailed understanding of the gallery system development patterns of *Ips hauseri*.

## 1. Introduction

Bark beetles (Coleoptera: Curculionidae: Scolytinae) represent one of the most ecologically significant and economically damaging groups of forest insects worldwide [1]. Among them, species of the *Ips* genus are particularly notorious for their capacity to inflict severe mortality on coniferous trees, altering forest structure, species composition, and succession dynamics [2,3]. These small, phloem-feeding beetles are primary consumers in conifer ecosystems, yet their transition from benign decomposers of weakened trees to aggressive primary pests is a phenomenon increasingly linked to global environmental change [4]. With 37 known species primarily distributed across Northern Hemisphere coniferous forests [5], *Ips* genus exhibit remarkable biological diversity. Their life cycle is tied to the phloem and cambial tissues of Pinaceae hosts—*Picea*, *Pinus*, and *Larix*—where they construct intricate gallery systems for mating, oviposition, and larval development [6]. These galleries disrupt the tree’s vascular system, leading to rapid decline and death, with annual global economic losses exceeding one billion U.S. dollars [7].

The ecological impact of *Ips* outbreaks extends far beyond individual tree mortality: by killing mature trees over vast areas, these beetles trigger landscape-level changes in carbon storage, hydrology, and biodiversity [3]. Recent decades have witnessed unprecedented outbreaks across the Northern Hemisphere, often amplified by climate anomalies. Europe’s 2018–2021 *Ips typographus* (Linnaeus, 1758) crisis destroyed millions of cubic meters of spruce forest, exposing the vulnerability of even-aged monocultures to climate-driven pest amplification [8]; similarly, warming-facilitated outbreaks of *Dendroctonus ponderosae* (Hopkins, 1902) have caused landscape-scale pine mortality in North America [4]. These events underscore a pressing need to understand the mechanistic links between beetle biology, forest management, and anthropogenic climate change.

Reproductive behavior and gallery construction are central to bark beetle population ecology [9]. Most *Ips* species are polygynous: a male attracts multiple females to a nuptial chamber, and each female excavates a radiating maternal gallery where she lays eggs in lateral niches [1]. Architectural complexity—measured by the number of maternal galleries (harem size), their length, orientation, and spatial arrangement—directly affects offspring number, larval competition, and survival [10]. Moreover, gallery construction is a plastic trait modulated by host vigor, phloem thickness, beetle density, and microclimate [11].

Recent research has revealed that reproductive strategies in Scolytinae may also be a key determinant of invasion potential. Dacquin et al. [12] demonstrated that pre-colonization mating—where females mate before dispersing to new host trees—is widespread among outbreeding species and is positively associated with a history of invasion. This trait allows a single mated female to establish a new population independently [13]. This finding adds a crucial behavioral dimension to the set of traits (e.g., polyphagy, association with symbiotic fungi, human-mediated dispersal) already linked to scolytine invasiveness [14]. How such mating strategies interact with gallery construction plasticity to influence outbreak dynamics in native and novel ranges remains an area of active research.

Within this context, *Ips hauseri* Reitter emerges as a species of growing concern. Endemic to the high-altitude *Picea schrenkiana* forests of the Tianshan Mountains in Central Asia (spanning Kazakhstan, Kyrgyzstan, and China), *I. hauseri* has long been considered a significant pest [15,16]. However, its status is shifting from a chronic, locally important pest to an increasingly frequent and severe outbreak agent, driven by recent climate warming and prolonged host stress. Recent years have seen severe outbreaks reported across Xinjiang, China, in regions including Hami, Changji, Urumqi, and Yili [17,18]. These outbreaks, resulting in extensive mortality of *P. schrenkiana*, are likely synergistically driven by climate warming, prolonged drought stress on host trees, and anthropogenic forest disturbances [19]. Notably, climate change may be altering the insect’s fundamental life history. While traditionally considered univoltine in high-altitude zones, evidence now suggests that under warmer conditions at elevations of 2000–2500 m, *I. hauseri* can complete three generations in two years, potentially accelerating population growth and outbreak frequency [19,20].

Despite its escalating impact, critical gaps persist in our knowledge of *I. hauseri*’s basic biology and ecology. It is known to be polyphagous within Pinaceae, attacking *Picea*, *Pinus*, and *Larix* [21], and older records suggest it typically constructs galleries with 3–5 maternal branches [16]. However, a comprehensive description of its gallery architecture—encompassing morphological diversity, dimensional metrics, and structural classification—is entirely lacking. Similarly, its reproductive behavior remains enigmatic. While conspecifics like *I. typographus* have been extensively studied (e.g., mating frequency, gallery initiation patterns) [22,23], parallel data for *I. hauseri* are absent.

Therefore, this study was conducted during a major *I. hauseri* outbreak in the *Picea schrenkiana* forests of Hami, Xinjiang, with three primary objectives: (1) to determine whether *I. hauseri* exhibits the longitudinal gallery pattern typical of *Ips* species and to provide the first detailed morphological classification and quantitative description of its gallery architecture (length, width, harem size); (2) to quantify the range of harem size and its relationship with reproductive output, specifically testing the effects of gallery orientation and spatial position on egg production and offspring development; and (3) to evaluate how host tree DBH modulates gallery complexity and to assess how these architectural traits mediate intraspecific competition and population dynamics under outbreak densities. By integrating detailed field dissections with quantitative analysis, this study bridges a critical knowledge gap and provides foundational insights into the reproductive ecology of *I. hauseri*, with direct implications for pest monitoring and broader theoretical frameworks linking insect behavior, reproductive strategy, and population dynamics under environmental change.

## 2. Materials and Methods

### 2.1. Study Area and Outbreak Context

The study was conducted in the *Picea schrenkiana* (Schrenk spruce) forest managed by the Xiheigou Forest Management Office in Hami City, Xinjiang Uygur Autonomous Region, China (Supplementary Materials Figure S1). The site is located on the northern slope of the eastern Tianshan Mountains (43°21′40″–43°34′1″ N, 92°30′25″–93°33′26″ E). This region has experienced a severe, ongoing outbreak of *Ips hauseri* since 2020. Fieldwork was carried out during the bark beetle activity seasons from May to September in 2024 and 2025, capturing two consecutive outbreak years.

The research area comprised four distinct sample plots (Table 1) established within the Xiheigou Forest Farm. The plots were situated in a middle mountain zone at elevations ranging from 2000 to 2500 m a.s.l. The climate is cold semi-arid, with a mean annual temperature of approximately 1 °C and annual precipitation of ~220 mm. The forest stand is dominated by mature *Picea schrenkiana* (80–120 years old, 10–12 m mean height), with scattered *Larix sibirica*. To capture a gradient of infestation pressure and stand conditions, plots were selected based on preliminary surveys:

Plots 1 and 2: Characterized by high, active *I. hauseri* population densities, evident from numerous newly attacked trees and fresh resinosis.

Plot 3: Represented a post-outbreak area where the main infestation wave had passed, leaving predominantly dead and decaying trees with low contemporary beetle activity.

Plot 4: A newly invaded area at the outbreak frontier, with early signs of infestation and low beetle density.

All plots were within a managed natural forest. Plot 3 was geographically separated from the others by a valley. Historical infestation patterns suggested the outbreak likely originated in Plot 3 before spreading to Plots 1, 2, and 4.

**Table 1:** Stand characteristics and infestation severity of the four sample plots in the Xiheigou Forest. Abbreviations: DBH (diameter at breast height).

Sample Plot	Coordinate	Elevation (m)	Slope	Slope Direction	Number of Plots	Canopy Density	DBH (cm)	Victimization Rates (%)
1	43°32′ N 92°55′ E	2201	0.46	North	5	moderate (60%)	14.7	53
2	43°32′ N 92°56′ E	2243	0.57	North	5	moderate (53%)	18.7	22
3	43°33′ N 92°57′ E	2335	0.76	North	3	sparse (43%)	14.3	80
4	43°32′ N 92°57′ E	2291	0.62	North	3	dense (68%)	16.1	9

### 2.2. Experimental Design and Sampling

The study combined two complementary sampling approaches to investigate gallery development: (1) using freshly felled trap logs to initiate and track synchronous gallery establishment, and (2) sampling naturally infested standing trees across a host diameter gradient to assess gallery patterns under field conditions.

#### 2.2.1. Trap Log Establishment and Monitoring

To obtain galleries of known and synchronized initiation time, we established trap logs. In late May 2024, coinciding with the peak spring emergence of overwintered *I. hauseri* adults, we selected three recently attacked *P. schrenkiana* trees (DBH: 14.2, 15.4, 17.6 cm) from Plots 1 and 2, based on the presence of fresh boring dust. These trees were felled, delimbed, and placed on the forest floor at mutually isolated locations (>100 m apart, exceeding the typical dispersal range of *I. hauseri* to ensure independent colonization events).

For 15 consecutive days post-felling, each log was inspected daily for new beetle entrance holes. Each new hole was immediately marked with a unique, waterproof code, and its location, date, and associated tree were recorded. This rigorous daily census allowed us to pinpoint the exact initiation date for every gallery subsequently sampled, a critical variable for developmental stage analysis.

#### 2.2.2. Sampling of Naturally Infested Standing Trees

To examine gallery architecture across a range of host sizes and infestation densities, we systematically sampled standing trees. In May 2024, three sampling transects were established within the infestation zones of Plots 1 and 4. Along each transect, five groups of standard trees were selected, with >50 m between groups to maintain spatial independence. Each group consisted of five *P. schrenkiana* trees that showed fresh signs of attack (e.g., new entrance holes, resin tubes) and exhibited declining vigor. In total, 15 groups (75 trees) were marked.

To assess the effect of host resource availability on gallery construction, we stratified sampling by tree diameter at breast height (DBH). We defined four DBH classes: 5 < d ≤ 10 cm, 10 < d ≤ 15 cm, 15 < d ≤ 20 cm, and 20 < d ≤ 25 cm. For each class, three sample trees were selected, and from each tree, ten non-overlapping, clearly discernible galleries within the 1–2 m height interval of the trunk were randomly chosen for dissection (Table 2). This resulted in 30 galleries per DBH class (120 galleries total from standing trees). Each selected gallery entrance was marked with colored paint corresponding to its sampling date.

#### 2.2.3. Gallery Dissection and Data Collection

Following the marking of a new entrance hole (on either trap logs or standing trees), the associated gallery was dissected at a predetermined developmental stage (Supplementary Materials Figure S2). A rectangular bark section (20 × 20 cm), centered on the entrance hole, was carefully removed using a chisel and knife to fully expose the phloem and the gallery system without damaging its structure. Consistent with the foraging behavior of *Ips* beetles, each entrance hole was assumed to correspond to one discrete gallery system for analysis.

Dissections were timed to capture three critical phases of gallery development, with a total of 219 gallery systems dissected over the two-year study period (2024–2025):

Early Phase (3–5 days post-colonization, n = 49, Supplementary Materials Figure S2c): Focused on initial gallery establishment. We recorded the number and sex of adults present (determined using a stereo microscope Leica MZ6, Leica Microsystems, Wetzlar, Germany), documented the structure of the nuptial chamber, and noted the initiation points and directions of maternal galleries. Adults were collected for further analysis.

Mid Phase (12–15 days post-colonization, n = 41, Supplementary Materials Figure S2d): Corresponding to completed egg-laying. We measured the final length (cm) and maximum width (mm) of each maternal gallery using digital calipers (Mitutoyo, Mitutoyo Corporation, Kawasaki, Japan, 0.01 mm precision). All egg niches along both sides of each maternal gallery were counted to determine total egg production per gallery and per gallery system. The position of each egg relative to the nuptial chamber was mapped.

Late Phase (20–25 days post-colonization, n = 129, Supplementary Materials Figure S2e): Focused on offspring development. We counted all larval galleries (daughter galleries) emanating from each maternal gallery. For each daughter gallery, we measured its total length and its width at both the start (near the egg niche) and the end (at the pupal chamber), using digital calipers. The presence of pupae or callow adults was recorded, and the pupation rate was calculated as (number of pupal chambers/total number of daughter galleries) × 100.

The first attacks were recorded on 4 June 2024, and gallery activity continued until 14 September 2024, reflecting the seasonal activity period of *I. hauseri* in this region.

#### 2.2.4. Gallery Morphology Classification

Based on the dissections, we developed a morphological classification system for *I. hauseri* gallery systems. Galleries were first categorized by harem size (i.e., the number of maternal galleries per system), labeled with Roman numerals I (1 gallery) through VII (7 galleries). Within each harem size category, distinct architectural patterns based on the relative orientation (upward, downward, or lateral) and arrangement of the maternal galleries were identified and assigned alphabetic suffixes (e.g., III-a, III-b). This yielded 11 unique gallery morphologies (Supplementary Materials Figure S3).

### 2.3. Statistical Analysis

All statistical analyses were performed using SPSS Statistics 27 and R software (v4.3.0) [24,25]. Graphs were created with GraphPad Prism 10.1.2 [26]. Data are presented as mean ± standard deviation (SD). Significance was accepted at *p* < 0.05.

Prior to pooling data, we tested for potential confounding effects of sampling plot and sampling method. No significant differences were detected among the four sample plots for any of the key response variables (harem size, maternal gallery length, egg number; one-way ANOVA, all *p* > 0.05), nor between the two sampling approaches (trap logs vs. naturally infested standing trees; independent-samples *t*-tests, all *p* > 0.05). Therefore, data from all plots and both sampling methods were pooled for all subsequent analyses.

Gallery morphology and host DBH: To test the effect of host tree DBH class on harem size and gallery type frequency, we used one-way ANOVA (after confirming homogeneity of variance with Levene’s test) followed by Tukey’s HSD post hoc test for pairwise comparisons. Proportional data (frequency of gallery types) were compared using Chi-square tests of independence.

Gallery dimensions and reproductive output: Differences in maternal gallery length and width among gallery types (I–VII) were analyzed using one-way ANOVA and Tukey’s HSD test. The effects of gallery direction (upward vs. downward) and positional context (central vs. flanking maternal galleries in systems with ≥3 galleries) on gallery length, width, and egg number were compared using independent samples *t*-tests or Mann–Whitney U tests if data violated normality (assessed via the Shapiro–Wilk test).

Relationship between gallery length and fecundity: We employed linear regression to model the relationship between maternal gallery length and the number of eggs laid. The overall fit was assessed using the coefficient of determination (R^2^), and the significance of the regression slope was tested with an F-test. Separate models were constructed for upward- and downward-developing galleries.

Offspring gallery development: The effects of maternal gallery direction (upward vs. downward) on daughter gallery length and width were analyzed using independent-samples *t*-tests, as the data met normality and homogeneity assumptions. For comparisons involving maternal gallery position (central vs. flanking), which involved only two groups, *t*-tests were also used after verifying normality. A generalized linear model (GLM) with a Poisson distribution was used to analyze factors affecting the total number of eggs per gallery system, with harem size, mean maternal gallery length, and host DBH class as predictors.

## 3. Results

### 3.1. Sampling Overview and Gallery Developmental Stages

**Figure 1:**
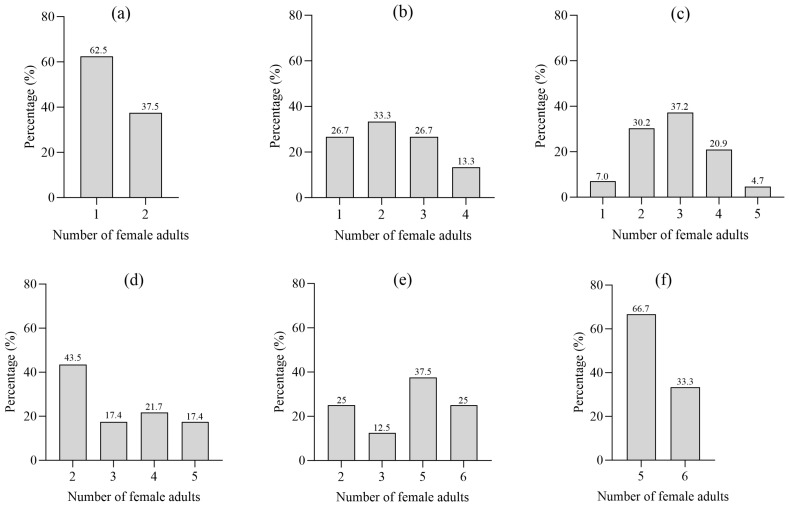
Gallery type classification and female-to-male ratio distribution. (**a**) Type I, (**b**) Type II, (**c**) Type III, (**d**) Type IV, (**e**) Type V, and (**f**) Type VI (*x*-axis: Different numbers of females per gallery within a specific gallery type; *y*-axis: Proportion of galleries with the same number of females within that gallery type). Note: Gallery types I–VI are classified based on the number of maternal galleries (i.e., harem size) within a gallery system. e.g., a Type I gallery system contains a single maternal gallery (harem size = 1). See Figure S3 for a detailed.

Over the two-year study period (2024–2025), we dissected a total of 219 *Ips hauseri* gallery systems within *Picea schrenkiana* forests. Female adults were present in 97 of the 219 galleries examined (44.3%), with 1 to 6 individuals per gallery and a mean of 2.9 ± 1.4 females per gallery. The distribution of female numbers varied among gallery types, with equal numbers of female insects observed in type I to VI galleries at rates of 62.5%, 33.3%, 37.2%, 21.7%, 37.5%, and 33.3% respectively. Type I, II, III, and V galleries exhibited the highest proportion of equal female insect numbers, contrasting with type IV and VI galleries (Figure 1).

### 3.2. Gallery Architecture and Morphological Classification

#### 3.2.1. Gallery Type Diversity and Harem Size Distribution

**Figure 2:**
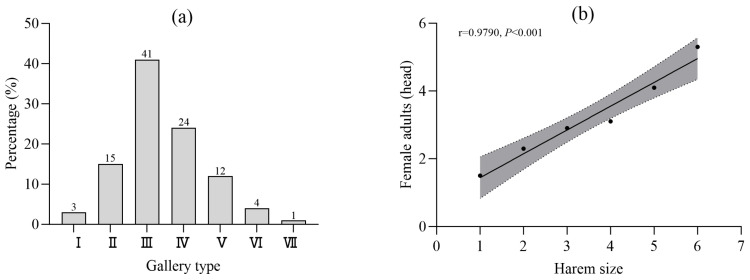
Gallery type classification and female distribution. (**a**) Proportion of various gallery types. Note: Gallery types I–VII are classified based on the number of maternal galleries (harem size); (**b**) Correlation between the quantity of adult female insects and the harem size (y = 0.70x + 0.74, *p* < 0.0001, R^2^ = 0.958).

We identified 11 distinct gallery morphologies based on maternal gallery arrangement and harem size (number of maternal galleries per system), ranging from Type I (1 gallery) to Type VII (7 galleries). Among these, Type III galleries (3 maternal galleries) were the most prevalent, constituting 41.1% of all systems, while Types II, III, IV, and V collectively accounted for 92.2% of observations. Type VII was exceptionally rare (1.4%) (Figure 2a).

Adult females were present in 44.3% of dissected systems (97 of 219). Harem size ranged from 1 to 6, with a mean of (2.9 ± 1.4) females per male. A strong positive linear relationship existed between the number of collected females and maternal gallery count per system (Linear regression: (y = 0.70x + 0.74, R^2^ = 0.958, *p* < 0.0001, F = 921.5, df = 95) (Figure 2b).

#### 3.2.2. Morphological Variations Within Gallery Types

**Figure 3:**
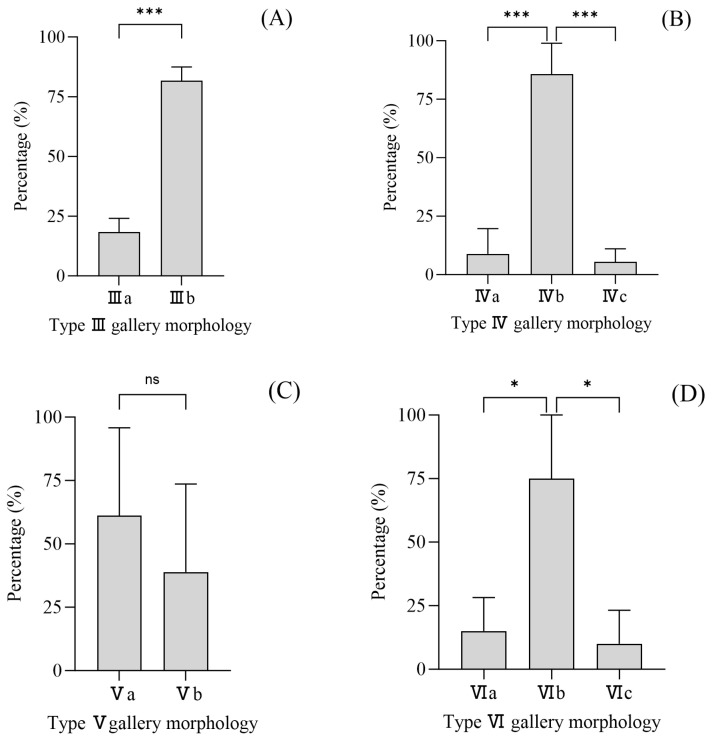
Morphological variation within gallery types. (**A**) Type III galleries: two distinct subtypes, III-a and III-b (χ^2^ = 26.1, df = 1, *p* < 0.001). (**B**) Type IV galleries: three subtypes, IV-a, IV-b, and IV-c (χ^2^ = 148.3, df = 2, *p* < 0.001). (**C**) Type V galleries: two subtypes, V-a and V-b (χ^2^ = 1.0, df = 1, *p* = 0.32). (**D**) Type VI galleries: three subtypes, VI-a, VI-b, and VI-c (χ^2^ = 45.0, df = 2, *p* < 0.001). Values are presented as the percentage of each subtype within the respective gallery type. (Statistical significance was assessed using Chi-square goodness-of-fit tests. Significant differences are shown by asterisks: ns = not significant results, * *p* < 0.05, *** *p* < 0.001). (Note: Gallery systems are categorized by the number of maternal galleries they contain, with each quantity assigned a corresponding Roman numeral: I, II, III, IV, V, VI, or VII. Within each type, specific morphologies are distinguished by the vertical orientation of the individual maternal galleries and are labeled sequentially with the letters a, b, c, and so forth. Consequently, each gallery system is assigned a unique alphanumeric code (Roman numeral + letter). For example, code “III a”: the numeral “III” indicates three maternal galleries within the system, and the letter “a” denotes one specific developmental morphology for systems with three galleries).

Detailed schematic representations of all 11 gallery morphologies are provided in Figure S3c. Significant morphological diversity existed within harem size categories. For example, Type III comprised two distinct shapes (III-a and III-b), with III-b being dominant (75.5%). Type IV included three shapes, of which IV-b was overwhelmingly prevalent (85.7%). Type V exhibited two shapes with no significant frequency difference, while Type VI showed a clear predominance of shape VI-b (75.0%) (Figure 3).

### 3.3. Influences of Host Tree Size on Gallery Structure

**Figure 4:**
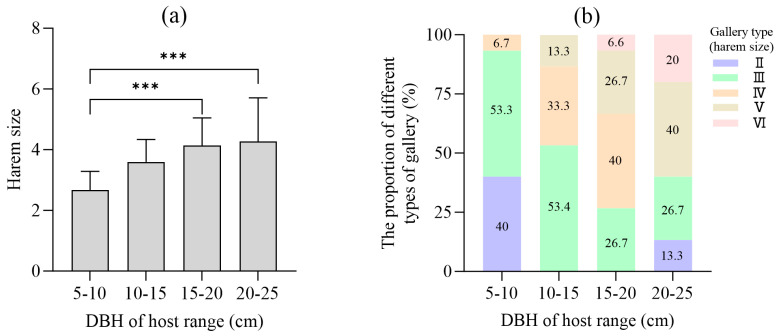
Influence of host tree DBH on gallery complexity. (**a**) Harem size on different host DBH (*p* < 0.01, R^2^ = 0.31); (**b**) Proportion of each gallery type on different host DBH. (One-way ANOVA, Tukey’s HSD post hoc test; significant differences are shown by asterisks: *** *p* < 0.001).

Host tree diameter at breast height (DBH) significantly influenced gallery complexity. Mean harem size increased with DBH: from (2.7 ± 1.1) (5–10 cm) to (4.3 ± 1.4) (20–25 cm) (One-way ANOVA: (F(_3,95_) = 8.37, *p* < 0.001, η^2^ = 0.21). Larger trees (DBH >15 cm) supported a greater diversity of gallery types and were predominantly colonized by Types IV and V, while smaller trees (5–15 cm DBH) primarily hosted Type III systems (Figure 4).

### 3.4. Gallery Dimensions and Orientation Effects

**Figure 5:**
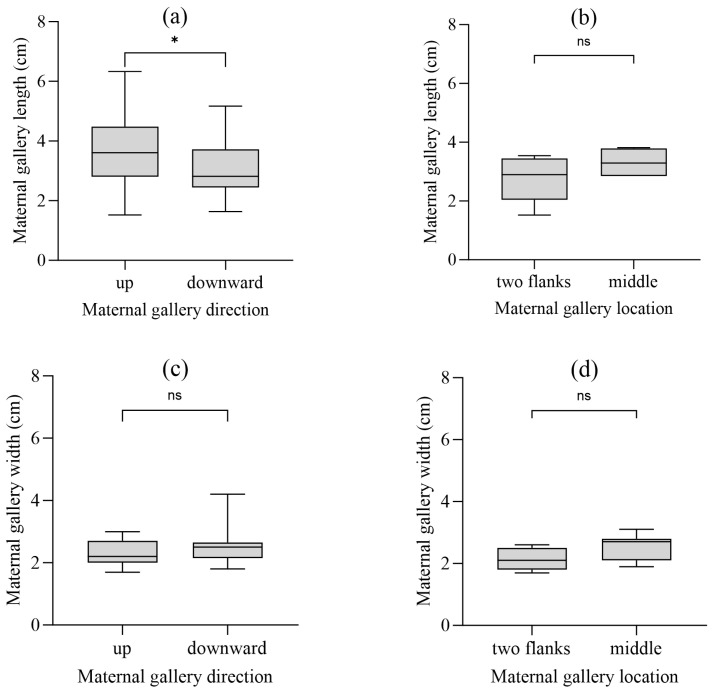
Dimensions of maternal galleries across orientations. (**a**,**c**) Length and width differences in upward vs. downward orientations; (**b**,**d**) Length and width differences in central vs. flanking orientations. (Independent-samples *t*-test; significant differences are shown by asterisks: ns = not significant results, * *p* < 0.05).

Across all systems, mean maternal gallery length was (3.4 ± 1.2) cm and width was (2.4 ± 0.5) mm (Table 3). Gallery dimensions varied significantly among types (One-way ANOVA for length: F(_6,212_) = 118.5, *p* < 0.001, η^2^ = 0.77; for width: F(_6,212_) = 15.8, *p* < 0.001, η^2^ = 0.31). Type I systems exhibited the longest galleries (6.12 ± 1.23 cm), while length progressively decreased with increasing harem size. Notably, upward-oriented galleries were significantly longer (3.79 ± 1.28 cm) than downward ones (3.10 ± 0.98 cm) (*t*-test: t = 4.89, df = 217, *p* < 0.001, Cohen’s d = 0.59) (Figure 5a,c). Within systems with ≥3 maternal galleries, central galleries tended to be shorter than flanking ones, though this difference was not statistically significant (*t*-test: t = 1.95, df = 62, *p* = 0.06) (Figure 5b,d).

### 3.5. Reproductive Output and Its Determinants

#### 3.5.1. Fecundity Across Gallery Types and Orientations

**Figure 6:**
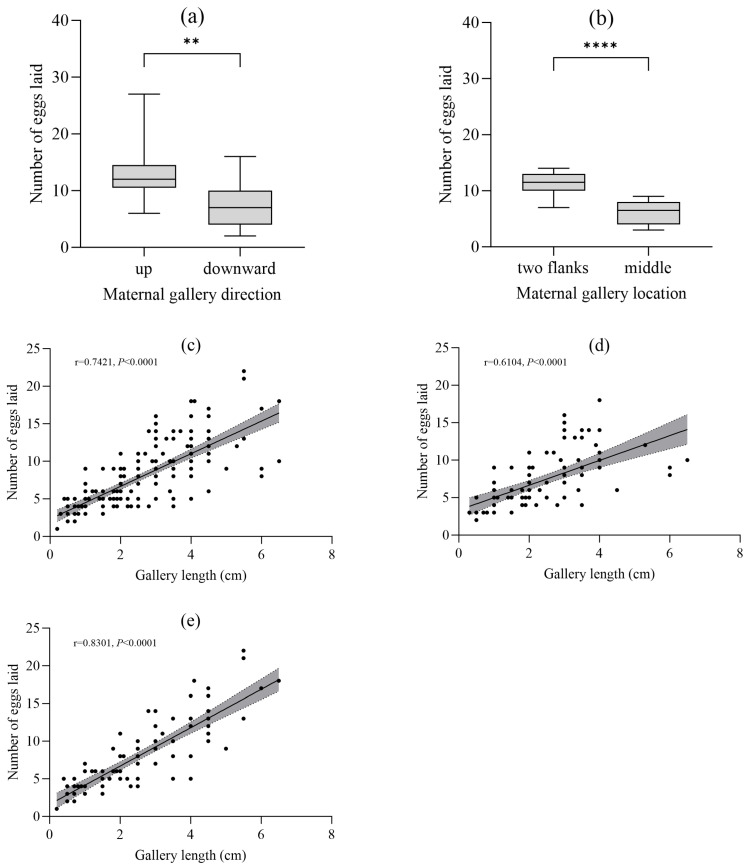
Reproductive output patterns. (**a**,**b**) Variation in spawning quantity concerning directions and positions of gallery (independent-samples *t*-test; significant differences are shown by asterisks: ** *p* < 0.01, **** *p* < 0.0001); (**c**–**e**) Correlation between gallery length and egg number in overall, upward, and downward galleries (Shaded bands indicate 95% confidence intervals. Regression equations, R^2^, *p*, and F statistics are shown within each panel).

The mean total fecundity per gallery system was 31.2 ± 14.5 eggs. Egg production peaked in Type V systems (46.9 ± 8.4 eggs) and declined in larger harems (Types VI and VII) (Table 3). Upward galleries contained nearly twice as many eggs as downward galleries (13.0 ± 4.9 vs. 7.1 ± 3.9; *t*-test: t = 8.52, df = 217, *p* < 0.001, Cohen’s d = 1.35), and within multi-gallery systems, central galleries received significantly fewer eggs than flanking galleries (6.0 ± 2.0 vs. 11.3 ± 2.0; *t*-test: t = 6.34, df = 75, *p* < 0.001, Cohen’s d = 2.65) (Figure 6a,b).

#### 3.5.2. Relationship Between Gallery Length and Egg Number

A significant positive linear relationship was found between maternal gallery length and egg number (Overall: y = 2.17x + 2.32, R^2^ = 0.55, *p* < 0.001, F = 260.8, df = 215). This relationship was stronger for downward galleries (R^2^ = 0.69, F = 178.2, df = 80) than upward ones (R^2^ = 0.40, F = 89.3, df = 134) (Figure 6c–e).

### 3.6. Offspring Development in Relation to Maternal Gallery Position

**Figure 7:**
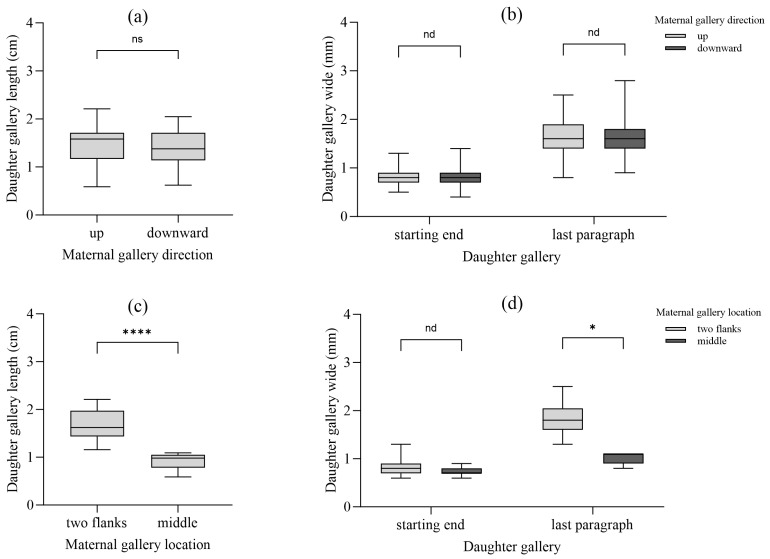
Offspring gallery development. (**a**,**b**) Length and width of daughter galleries by maternal orientation; (**c**,**d**) Length and width of daughter galleries by maternal position (central vs. flanking). Note: Data labeled with statistical significance (*p*-values and effect sizes). (Independent-samples *t*-test; significant differences are shown by asterisks: ns = not significant results or nd = not a discovery, * *p* < 0.05, **** *p* < 0.001).

Each system produced an average of 22.8 ± 7.3 daughter galleries, with a mean length of 1.44 ± 0.36 cm. Larval gallery width increased from 0.80 ± 0.18 mm initially to 1.64 ± 0.35 mm terminally (Table 4). Pupation rate was 85.2%. While maternal gallery orientation did not significantly affect daughter gallery development, horizontal position had a strong influence: daughter galleries from flanking maternal galleries were significantly longer and wider than those from central galleries (Length: 1.66 ± 0.29 cm vs. 0.92 ± 0.16 cm, *t*-test: t = 9.87, df = 58, *p* < 0.001, Cohen’s d = 2.98; Terminal width: 1.84 ± 0.31 mm vs. 1.02 ± 0.11 mm, *t*-test: t = 8.95, df = 58, *p* < 0.001, Cohen’s d = 3.42) (Figure 7).

## 4. Discussion

### 4.1. Gallery Architecture Diversity and Its Ecological Significance in Ips hauseri

Our study provides the first comprehensive documentation of gallery architecture in *Ips hauseri*, revealing remarkable morphological diversity and adaptive complexity. We identified 11 distinct gallery morphologies, with harem sizes ranging from 1 to 7 maternal galleries. This observed range (1–7) extends beyond the previously reported 3–5 maternal galleries for this species [16], suggesting either regional variation or, more likely, an undocumented behavioral plasticity in response to outbreak conditions. Such plasticity may represent a critical adaptive strategy enabling *I. hauseri* to exploit heterogeneous resource environments within host trees and mitigate intense intraspecific competition under high population densities—a phenomenon noted in other bark beetle systems during eruptions [4]. Our observation extends that reported by Parfentieva [27] in the 1950s. This discrepancy may reflect geographic or temporal variation, an outbreak-induced shift toward larger harems, or our more intensive sampling and quantitative approach. Parfentieva’s foundational description lacked detailed morphometrics and ecological analyses; our study therefore both corroborates and substantially expands those early findings by providing the first comprehensive, quantitative characterization of gallery architecture and its determinants in *I. hauseri*.

All gallery systems conformed to the longitudinal structural type characteristic of the genus *Ips* [28], exhibiting the classic pattern of dispersed oviposition along maternal galleries. Within this framework, however, *I. hauseri* exhibited greater architectural complexity than its well-studied congener, *I. typographus*. While *I. typographus* typically produces systems with 2–3 maternal galleries [23,29], which correspond to our Type II and III morphologies, *I. hauseri* frequently constructed systems with 4 or 5 maternal galleries (Types IV and V). This suggests that under similar ecological constraints, *I. hauseri* may employ a strategy of horizontal resource exploitation, constructing more but shorter maternal galleries when vertical space is limited, thereby accommodating more females per male and potentially maximizing the use of available phloem area. This aligns with the concept that gallery architecture is not merely a taxonomic trait but a functional adaptation to local resource distribution and competition pressure [5].

An intriguing finding was the relationship between harem size (odd vs. even) and gallery symmetry. Systems with even-numbered harem sizes (II, IV, VI) exhibited a strong tendency towards symmetrical, balanced arrangements of maternal galleries (e.g., our dominant IV-b and VI-b morphologies). In contrast, odd-numbered systems (III, V, VII) displayed more variable, often asymmetrical configurations. This pattern implies a potential behavioral rule or physical constraint during gallery initiation, possibly related to the spatial negotiation among females or a male-mediated spacing mechanism to reduce immediate interference. While such a pattern has not been explicitly reported in other *Ips* species, it underscores the sophistication of collective construction behavior in bark beetles and warrants further ethological and perhaps computational modeling investigation.

### 4.2. Mating System, Operational Sex Ratio, and the Harem Size Proxy

Bark beetles predominantly exhibit either monogynous or polygynous mating systems [9]. Our results confirm that *I. hauseri* is polygynous, with a single male mating with multiple females. The operational sex ratio, indicated by the number of females per gallery system, varied from 1 to 6, demonstrating considerable flexibility. This range is comparable to, though potentially more variable than, that of *I. typographus*, which commonly has 2–3 females per male [30]. The strong positive correlation we found between the number of collected females and the number of maternal galleries validates the use of “harem size” (i.e., maternal gallery count) as a reliable, field-observable proxy for the operational sex ratio. This is a significant practical finding, as directly observing mating events or collecting all adults from a gallery is often difficult. Schlyter and Zhang [31] similarly used gallery structure to infer mating patterns in other *Ips* species. Our work extends this approach to *I. hauseri*, providing a non-invasive tool for estimating a key demographic parameter—the number of breeding females per male—which is central to understanding population growth potential and Allee effects during colonization [13].

The ability of a single male to secure and mate with up to six females suggests effective male aggregation pheromones and/or a scarcity of males relative to receptive females under outbreak conditions. This high variance in female number per male also links directly to the observed gallery diversity. More females necessitate more maternal galleries, but as our data show, this comes at a cost to individual gallery development, setting up a critical trade-off.

### 4.3. Reproductive Trade-Offs: Harem Size, Gallery Investment, and Optimal Fecundity

A central finding of this study is the demonstration of a clear trade-off between harem size and individual reproductive investment in *I. hauseri*. While total egg output per gallery system increased with harem size up to a point, the fecundity per female (eggs per maternal gallery) declined. This was mechanistically driven by the reduction in individual maternal gallery length as harem size increased. The positive correlation between gallery length and egg number is a fundamental constraint; longer galleries provide more niches for eggs. Consequently, in systems with many females, each female excavates a shorter gallery, laying fewer eggs.

This trade-off culminated in an optimal harem size for total system fecundity at five maternal galleries (Type V), which produced the highest mean number of eggs (~47). This optimum represents a balance between adding more egg-laying females and diminishing returns from increased competition among them. Remarkably, a similar optimal harem size (four galleries) was reported for *Ips grandicollis* by Latty et al. [10], who also attributed the decline beyond the optimum to intensified female-female competition for space and male attention. Our results for *I. hauseri* thus fit a broader pattern within the genus, suggesting a general evolutionary constraint on polygyny in phloem-feeding bark beetles: while attracting multiple females increases a male’s reproductive potential, severe intraspecific competition among his mates can reduce the success of each to the point of lowering overall reproductive yield.

The mean total fecundity of *I. hauseri* (31 eggs/system) was lower than that reported for *I. typographus* (often 60–80 eggs/system) [23]. This can be largely explained by the shorter maternal galleries of *I. hauseri* (3.4 cm vs. 8–10 cm in *I. typographus*) [32]. Several factors may underlie this difference in excavation capacity: (1) Body size: *I. hauseri* adults in our study averaged 3.5–4 mm, slightly smaller than the 4–5 mm typical of *I. typographus* [16]. The body length of female individuals within specific galleries was not quantified in this study; only estimates based on a limited number of adults were provided. Smaller body size may limit physical excavation capability, but this remains a hypothesis that requires direct testing. (2) Outbreak density: The high population density during our study likely intensified competition for phloem thickness and quality, leading to shorter galleries—a density-dependent effect well-documented in bark beetles [33]. In *I. typographus*, increasing colonization density strongly reduces gallery length, per-capita oviposition, and offspring survival. Thus, density-driven suppression likely accounts for much of the observed fecundity gap. (3) Host condition: *Picea schrenkiana* under drought stress may have thinner or lower-quality phloem, constraining gallery expansion compared to healthier hosts typically exploited by *I. typographus* in its native range. *I. typographus* outbreaks typically occur in more productive, mesic Norway spruce forests with thicker phloem [32]. Although we did not measure phloem thickness directly, poor host condition probably exacerbates the fecundity difference. (4) Natural enemies and competitors. Enemy pressure can substantially reduce bark beetle reproductive success [1,4]. We observed occasional predators in our dissections, but their impact on *I. hauseri* remains unknown. Whether enemy pressure differs between Central Asian and European spruce forests is a critical knowledge gap. (5) Evolutionary history. As an endemic species in stable high-altitude forests, *I. hauseri* may have evolved a lower-fecundity, persistent population strategy, whereas *I. typographus* is adapted to disturbance-prone boreal systems where high reproductive output confers an advantage [4,20]. This hypothesis awaits comparative life-history studies.

### 4.4. The Interplay of Host Traits, Colonization Density, and Intraspecific Competition

Our results underscore that gallery development is not an intrinsic property of the beetle alone but is shaped by a dynamic interaction with the host tree and the beetle population itself. Host DBH was a major driver of gallery complexity. Larger trees (DBH > 15 cm) supported significantly larger harems and more complex gallery types (IV and V). This “host-size effect” is consistent across many bark beetle species [4,34]. Larger trees offer a greater quantity and potentially higher quality of phloem, providing more space and resources for a larger beetle cohort, thereby reducing immediate resource competition and allowing for the development of more extensive gallery systems. This creates a positive feedback loop: beetles preferentially attack larger, more resource-rich trees [4], which in turn support higher colonization densities and greater reproductive output, fueling population growth.

However, colonization density itself is a double-edged sword. While initial attack density on a suitable large host may be high, our data on offspring development reveal the severe consequences of within-gallery competition. In systems with multiple parallel maternal galleries, the centrally located gallery suffered a substantial competitive disadvantage. Females oviposited significantly fewer eggs in these central galleries, and the resulting larvae constructed shorter and narrower daughter galleries (Figure 7, Table 4). Although we did not directly observe agonistic interactions among larvae (e.g., physical combat or cannibalism), the pronounced differences in daughter gallery dimensions between central and flanking positions provide strong indirect evidence of competitive suppression. This is a direct manifestation of scramble competition for space and food, where offspring in central positions are squeezed from both sides. Schlyter and Zhang [31] observed a similar oviposition bias in *I. typographus*, with females avoiding central competitive zones. Kirkendall identified within-harem larval competition as a key, yet often overlooked, component of density-dependent mortality [35]. Our findings provide strong empirical support for this, demonstrating how maternal oviposition strategy (biasing effort toward flanking galleries) is a behavioral adaptation to pre-emptively mitigate competition among offspring.

An important question is what determines which females occupy the more favorable flanking positions. Several non-exclusive mechanisms may operate: (i) arrival order, with earlier females selecting superior sites; (ii) male mate choice or spacing behavior; (iii) female–female competition, where larger or more aggressive individuals secure preferred locations. Our data do not directly resolve these factors, but the strong correlation between female number and maternal gallery count (Figure 2b) suggests that males do not limit female access. Controlled experiments manipulating arrival sequence and female traits are needed to disentangle these mechanisms.

Interestingly, the vertical orientation of the gallery (upward vs. downward) also influenced reproductive investment, with upward galleries being longer and containing more eggs. This may relate to gravitational effects on resin flow, microclimate (temperature, moisture gradients), or simply the energetics of excavation. However, this orientation effect was secondary to the powerful effect of horizontal spatial position (central vs. flanking), which had a more dramatic impact on both egg number and subsequent larval development.

### 4.5. Behavioral Plasticity, Adaptive Strategies, and Implications for Invasiveness

The observed plasticity in gallery architecture—varying harem size, adjusting gallery length, and strategic oviposition—highlights *I. hauseri*’s behavioral adaptability. This plasticity is a critical component of its ecology, allowing it to optimize reproductive output across variable host conditions and population densities. By constructing more, shorter galleries on smaller hosts or under high density, the species maximizes the number of breeding females even when individual output is low—a strategy that may favor population persistence and growth in resource-limited or highly competitive environments.

This behavioral flexibility invites comparison with traits known to facilitate invasiveness in other Scolytinae. A recent pan-continental study by Dacquin et al. found that pre-colonization mating (females mating before finding a new host) is common in outbreeding species and is positively associated with a history of invasion [12]. While we did not directly test for pre-colonization mating in *I. hauseri*, its demonstrated plasticity in gallery establishment and the ability of a single mated female to initiate a gallery (as in Type I systems) suggest a capacity for independent foundation. If future research confirms that *I. hauseri* females frequently mate before dispersal, this would significantly elevate their potential risk as an invasive species. The ability to adjust gallery strategy based on resource availability further enhances its potential to establish in novel environments with different host tree sizes and conditions.

Furthermore, the outbreak dynamics of *I. hauseri* in the Tianshan region, likely accelerated by climate warming [19], mirror the patterns seen with invasive bark beetles elsewhere. The interaction between behavioral plasticity (allowing exploitation of stressed trees) and climate-driven host susceptibility creates a dangerous synergy that can lead to landscape-scale mortality.

### 4.6. Management Implications and Future Research Directions

The concealed nature of bark beetle damage within the phloem layer makes direct observation of their life cycle challenging, often delaying detection until tree mortality is irreversible [28]. Addressing these knowledge gaps is therefore not merely an academic exercise but a pressing management imperative. Our findings have direct implications for monitoring and managing *I. hauseri* populations. The number of maternal galleries (harem size) and the prevalence of specific gallery morphologies (especially Types IV and V) could serve as useful indicators of population pressure and reproductive intensity. Forest managers conducting post-outbreak surveys could use these gallery traits, easily observable from bark samples, to assess whether the local beetle population is in a growth phase (dominance of high-fecundity gallery types on large trees) or a declining phase (more simple galleries, smaller harems).

Future research should pursue several promising avenues. Molecular analysis of kinship using microsatellite markers to determine the relatedness of females within a harem and the paternity of offspring would clarify the mating system, test for multiple mating by females, and reveal the extent of inbreeding or outbreeding. Controlled experiments on logs with manipulated colonization densities would allow precise quantification of the competitive thresholds and their effects on gallery development and reproductive success. Investigation of pre-colonization mating by dissecting pre-dispersal females to determine sperm presence would test whether *I. hauseri* employs this strategy, directly linking our findings to the framework of invasion biology [12]. Finally, long-term studies correlating inter-annual climate variables (e.g., temperature, drought indices) with gallery morphology and fecundity would elucidate how climate change directly influences reproductive behavior and outbreak potential.

## 5. Conclusions

In conclusion, this study reveals that *Ips hauseri* possesses a complex and plastic gallery construction strategy finely tuned to navigate the trade-offs between harem size, individual reproductive investment, and intense intraspecific competition. Its reproductive optimum, host-size dependency, and strategic oviposition patterns are key adaptations to its outbreak ecology in Central Asian spruce forests. Understanding these behavioral mechanisms is crucial for predicting its population dynamics under ongoing climate change and for assessing its potential to threaten coniferous forests beyond its current range.

## Figures and Tables

**Table 2:** DBH classes and individual tree DBH values for gallery sampling.

DBH Range (cm)	DBH of Sample Plant (cm)
5 < d ≤ 10	8.7, 9, 9.5
10 < d ≤ 15	11.6, 12.6, 14.9
15 < d ≤ 20	15.7, 16.8, 19.6
20 < d ≤ 25	20.5, 21.3, 22

**Table 3:** Measurements of mean maternal gallery dimensions and fecundity across gallery types.

Gallery Type	Gallery Length (cm)	Gallery Width (mm)	Number of Eggs Laid
I	6.12 ± 1.23 a	2.57 ± 0.38 a	9.29 ± 2.96 c
II	4.46 ± 0.58 b	2.57 ± 0.54 a	20.13 ± 7.4 b
III	3.46 ± 0.56 c	2.46 ± 0.33 a	26.34 ± 9.16 b
IV	3.79 ± 0.98 bc	2.51 ± 0.42 a	36.78 ± 11.97 a
V	2.46 ± 0.69 d	1.99 ± 0.24 b	46.91 ± 8.42 a
VI	1.79 ± 0.29 d	1.93 ± 0.27 b	36.86 ± 11.76 a
VII	1.64 ± 0.18 d	1.75 ± 0.17 b	24.5 ± 4.5 b

Note: Values are presented as mean ± standard deviation. Different lowercase letters within the same column indicate statistically significant differences among gallery types (one-way ANOVA followed by Tukey’s HSD post hoc test, *p* < 0.05). Groups sharing the same letter are not significantly different.

**Table 4:** Presents diverse orientations and comprehensive developmental indices of the gallery structure in the bark beetle.

Gallery		Maternal Gallery		Egg	Daughter Gallery		
		Long (cm)	Wide (mm)	Number of Eggs	Long (cm)	Start Width (mm)	Terminal Width (mm)
Direction	Up	3.79 ± 1.28	2.34 ± 0.39	13.0 ± 4.91	1.81 ± 0.29	0.76 ± 0.15	1.64 ± 0.36
	Downward	3.1 ± 0.98	2.52 ± 0.52	7.1 ± 3.9	1.71 ± 0.32	0.78 ± 0.17	1.56 ± 0.27
Location	Two flanks	2.67 ± 0.69	2.11 ± 0.31	11.3 ± 2.01	1.66 ± 0.29	0.8 ± 0.19	1.84 ± 0.31
	Middle	3.3 ± 0.42	2.54 ± 0.4	6.0 ± 2.02	0.92 ± 0.16	0.73 ± 0.08	1.02 ± 0.11
Population mean		3.4 ± 1.2	2.41 ± 0.47	9.4 ± 4.53	1.44 ± 0.36	0.8 ± 0.18	1.64 ± 0.35

Note: The metrics for maternal gallery and daughter gallery are assessed post the finalization of gallery maturation, with egg quantities denoting the reproductive output of an individual maternal gallery.

## Data Availability

The raw data supporting the conclusions of this article will be made available by the authors on request.

## Supplementary Materials

The following supporting information can be downloaded at https://www.mdpi.com/article/10.3390/insects17030238/s1. Figure S1. Forest sections of different degrees of damage; Figure S2. Development diagram of the gallery system at different stages with *Ips hauseri* colonization; Figure S3. Schematic diagram of the different gallery systems of the *Ips hauseri.*
