# Supplementary material for: Gallery Architecture and Reproductive Strategy of Ips hauseri (Coleoptera: Curculionidae) in a Picea schrenkiana Forest: Implications for Population Dynamics Under Outbreak Conditions
Source: Insects. 2026 Feb 25;17(3):238. doi: 10.3390/insects17030238 (PMC13026913; doi:10.3390/insects17030238)
Supplement: Supplementary file 1 [file insects-17-00238-s001.zip › insects-4134326-supplementary.pdf]

Supplementary material

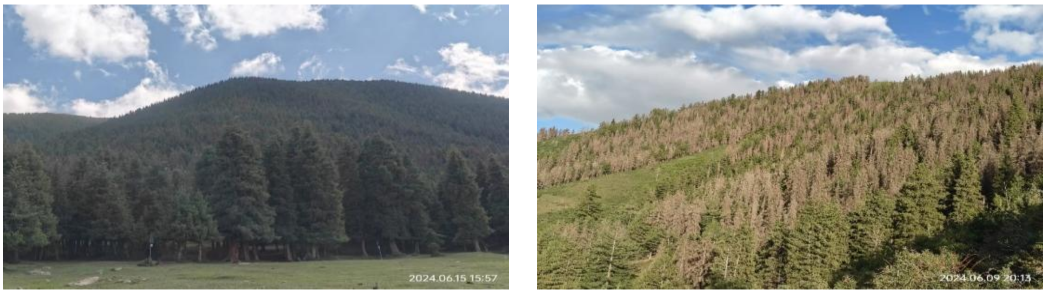

Figure S1 Forest sections of different degrees of damage (Research location: the Xiheigou Forest Management Office in Hami City, Xinjiang Uygur Autonomous Region, China; Type of vegetation: mixed spruce-larch forest).

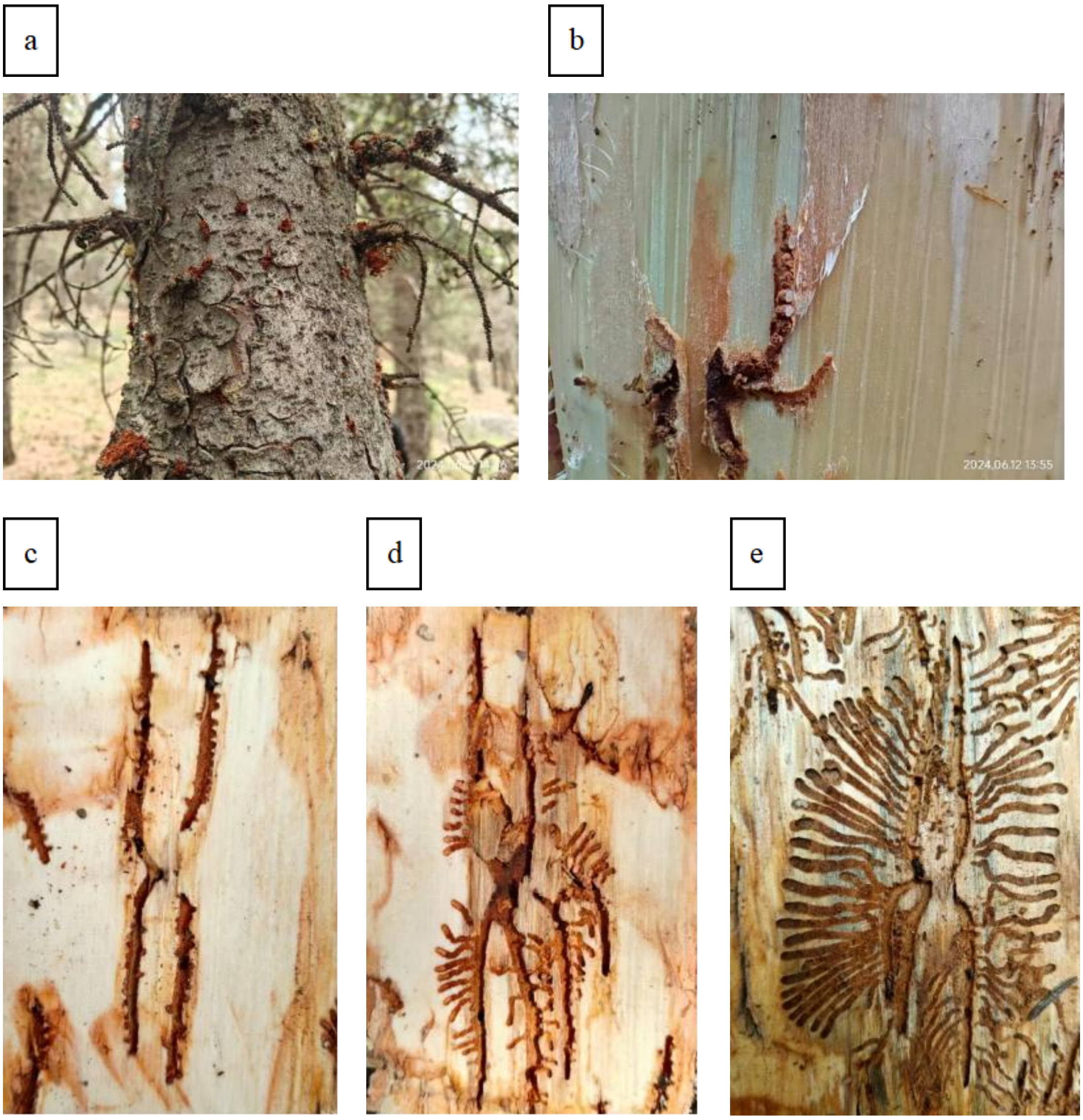

Figure S2 Development diagram of the gallery system at different stages with *Ips hauseri*

colonization (a. *Picea schrenkiana* standing trees newly colonized by *Ips hauseri*; b. Traces and residual eggs associated with *Ips hauseri* gallery construction; c. Gallery of *Ips hauseri* at the early stage of construction; d. Gallery of *Ips hauseri* at the middle stage of construction; e. Gallery of *Ips hauseri* at the late stage of construction).

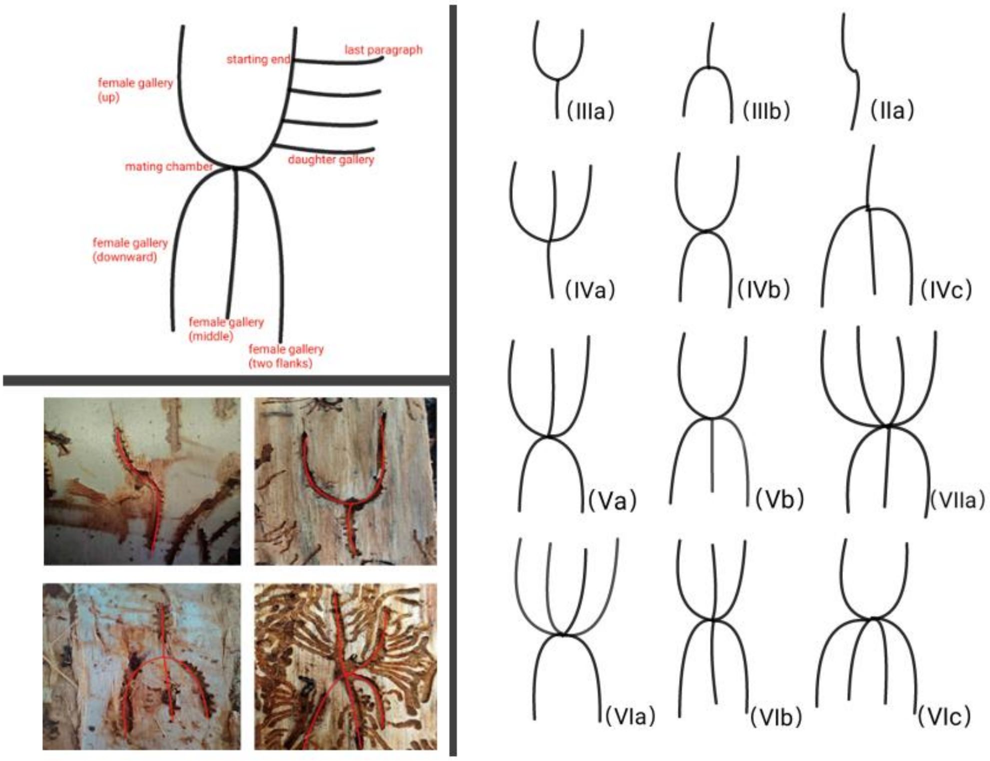

Figure S3 Schematic diagram of the different gallery systems of the *Ips hauseri*

(Note: Gallery systems are categorized by the number of maternal galleries they contain, with each quantity assigned a corresponding Roman numeral: I, II, III, IV, V, VI, or VII. Within each type, specific morphologies are distinguished by the vertical orientation of the individual ma-ternal galleries and are labeled sequentially with the letters a, b, c, and so forth. Consequently, each gallery system is assigned a unique alphanumeric code (Roman numeral + letter). For ex-ample, code "III a": the numeral "III" indicates three maternal galleries within the system, and the letter "a" denotes one specific developmental morphology for systems with three galleries).
